# Genetics‐based methods for agricultural insect pest management

**DOI:** 10.1111/afe.12241

**Published:** 2017-06-21

**Authors:** Nina Alphey, Michael B. Bonsall

**Affiliations:** ^1^ Mathematical Ecology Research Group, Department of Zoology, South Parks Road Oxford OX1 3PS U.K.; ^2^ Department of Life Sciences Imperial College London, Silwood Park Campus, Buckhurst Road Ascot SL5 7PY U.K.; ^3^ The Pirbright Institute, Ash Road Pirbirght GU24 0NF U.K.; ^4^ St Peter's College, New Inn Hall Street Oxford OX1 2DL U.K.

**Keywords:** Bt crops, genetic insect control, resistance management, self‐limiting constructs, sterile insect technique

## Abstract

1. The sterile insect technique is an area‐wide pest control method that reduces agricultural pest populations by releasing mass‐reared sterile insects, which then compete for mates with wild insects. Contemporary genetics‐based technologies use insects that are homozygous for a repressible dominant lethal genetic construct rather than being sterilized by irradiation.
2. Engineered strains of agricultural pest species, including moths such as the diamondback moth Plutella xylostella and fruit flies such as the Mediterranean fruit fly Ceratitis capitata, have been developed with lethality that only operates on females.
3. Transgenic crops expressing insecticidal toxins are widely used; the economic benefits of these crops would be lost if toxin resistance spread through the pest population. The primary resistance management method is a high‐dose/refuge strategy, requiring toxin‐free crops as refuges near the insecticidal crops, as well as toxin doses sufficiently high to kill wild‐type insects and insects heterozygous for a resistance allele.
4. Mass‐release of toxin‐sensitive engineered males (carrying female‐lethal genes), as well as suppressing populations, could substantially delay or reverse the spread of resistance. These transgenic insect technologies could form an effective resistance management strategy.
5. We outline some policy considerations for taking genetic insect control systems through to field implementation.

## Introduction

Many insects in agro‐ecosystems are considered to be major global pests causing significant economic harm. For example, the pink bollworm *Pectinophora gossypiella* (Saunders), a specialist pest of cotton, originated in Asia and spread to America, Australasia and Africa in the 20th Century (Naranjo *et al*., 2002). It is now present in almost all cotton‐growing countries, and is a key pest in many of them. The Mediterranean fruit fly (‘Medfly’) *Ceratitis capitata* (Wiedemann) is a highly invasive generalist attacking more than 250 host plants, and is one of the world's most economically important pests (CABI, 2016). Diamondback moth *Plutella xylostella* (L.), a pest of brassicas (including a number of vegetable and oilseed crops), has evolved resistance to all classes of synthetic insecticides, as well as to some biopesticides; it was the first insect observed to evolve field resistance to dichlorodiphenyltrichloroethane and to Bt (a biopesticide derived from a bacterium *Bacillus thuringiensis*). Diamondback moth costs the global economy an estimated US$4–5 billion per year through a combination of lost yield and costs of management (Zalucki *et al*., 2012; Furlong *et al*., 2013).

Conventional control methods, particularly chemical insecticides, have often failed to prevent the enormous damage caused by insect pests, and advances in biology (rather than chemistry) have been harnessed to provide novel control options. One alternative against diamondback moth is Bt biopesticide in the form of sprays (Furlong *et al*., 2013). Genetically modified (GM) insecticidal crops express these Bt toxins to protect the plant from target pests. For example, Bt cotton defends against Lepidoptera, including pink bollworm (Carrière *et al*., 2003), by expressing Cry 1Ac toxins, which are specifically lethal to Lepidoptera (de Maagd *et al*., 2001). An area‐wide method known as the sterile insect technique (SIT) has been very successful against pink bollworm and Medfly (Dyck *et al*., 2005) and is being improved using advances in molecular biology. For all three of these example species, new genetic insect control methods are being developed to tackle agriculturally important pest populations. In this review, we set out an overview of genetic insect control methods and, in doing so, we give an indication of how mathematical modelling is useful in providing insights (and exploring limitations) to these technologies in the absence of broad evidence from experimental field trials and observations. This area of research is highly interdisciplinary; our focus is on theoretical analysis, considering ecology and genetics together to help design, understand, test and implement these novel strategies for agricultural insect pest management.

## Sterile insect methods

The idea of releasing sterile insects into wild populations as a pest management intervention was independently conceived in the 1930s and 1940s by geneticist A. S. Serebrowskii in Moscow; tsetse field researcher F. L. Van der Planck in what is now Tanzania; and E. F. Knipling at the U.S. Department of Agriculture (USDA) (Klassen & Curtis, 2005). Van der Planck and Serebrowskii focussed on sterility resulting from hybrid crosses between different species or different genetic strains. Knipling (1955) pursued the use of ionizing radiation to induce dominant lethal mutations causing sterility.

In current practice, the SIT involves the mass rearing of the pest species on artificial diet, exposing very large batches of individuals to radiation to cause chromosome damage, followed by their release into a target area. When the released insects mate, the resulting eggs do not hatch because of the damage to genetic material in the parent's germ line. Sustained inundative releases are required. Sufficient sterile insects must be released for a long enough period to achieve a significant reduction in pest numbers, either suppression to a suitably low density or local population elimination. One important measure is a release ratio, or over‐flooding ratio, of released sterile insects to wild fertile insects. The SIT is mating‐based, relying on biology rather than chemistry to tackle pest populations. It is species‐specific and so has no direct off‐target effects on other species in the environment, and is best‐suited to systems where a single species is the major cause of harm.

Area‐wide SIT programmes have achieved success on very large scales (Dyck *et al*., 2005). Decades‐long international campaigns have suppressed and eradicated the New World screwworm *Cochliomyia hominivorax* (Coquerel) from the U.S.A., Mexico, Belize, Guatemala, Honduras, El Salvador, Nicaragua, Costa Rica, Panama and some Caribbean islands (Vargas‐Teran *et al*., 2005). Continuing releases in Panama form a barrier to prevent reinvasion into Central and North America from South America. SIT was also deployed against a screwworm outbreak in Libya. Screwworm is a myiasis pest whose larvae develop in living tissue of vertebrates, notably cattle, but also other livestock, wildlife and occasionally humans (e.g. in wounds). Freedom from infestation in those countries has produced significant economic benefits that vastly outweigh the costs of intervention (Vargas‐Teran *et al*., 2005).

SIT programmes typically release both males and females, lacking a practical method to sort the sexes easily in large numbers. This is inefficient because the released sterile females and males tend to court and mate with each other rather than seeking out wild mates. Male‐only releases are generally more efficient than mixed sex releases, a large‐scale study of irradiated Medfly quantified this as being three‐ to five‐fold more efficient per male (Rendón *et al*., 2004). Early removal of females (eggs or early larval instars) in the generation destined for release also potentially saves on rearing costs as only the males need to be housed and fed.

**Figure 1:**
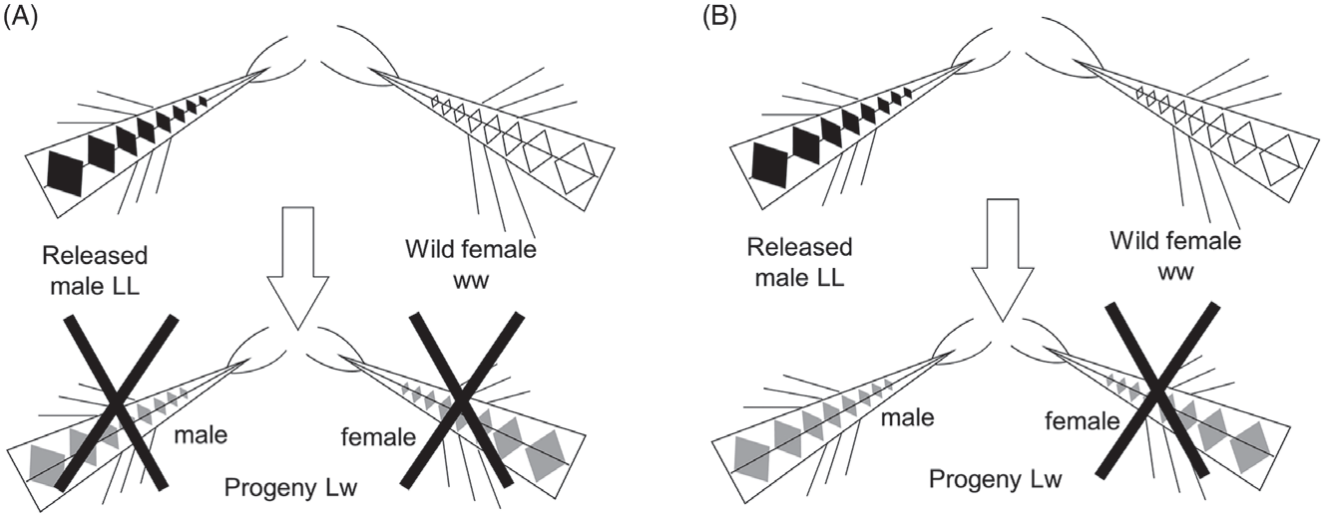
Genetics. Engineered males carry two copies of a genetic construct ‘L’, which is a repressible dominant lethal. The wild‐type allele ‘w’ represents the absence of the engineered construct. All offspring of released males and wild females inherit one copy of the dominant lethal. In (A), those progeny are not viable. In (B), the engineered lethality is female‐specific; thus, female progeny are nonviable and male progeny survive and can pass on the construct to half of their own offspring.

Genetics‐based variants of the SIT are being developed (Thomas *et al*., 2000; Alphey, 2014; Alphey & Alphey, 2014; Alphey *et al*., 2014). Various insect species, crop pests and human disease vectors are undergoing trials ranging from laboratory experiments to large‐scale open releases (Gong *et al*., 2005; Ant *et al*., 2012; Harris *et al*., 2012; Harvey‐Samuel *et al*., 2015). Insects have been engineered with a self‐limiting construct conferring a dominant lethal phenotype. Male insects that are homozygous for that transgene are released to mate with wild females, whose progeny inherit the dominant lethal and so are unable to survive to reproductive maturity (Fig. 1A). The population‐level outcome is then identical to SIT: a reduction in population size.

To allow such insect strains to be produced and mass reared, the construct is repressible. The laboratory or factory diet contains an antidote that switches off expression of the lethal effector gene. In the simplest version, this works in a similar manner to radiation‐based SIT. Compared with sterilizing doses of radiation, the targeted nature of genetic engineering generally mitigates fitness reductions in transgenic insects; although some detriment in performance might occur, there will be little or no effect in the most promising candidate genetic lines (Marrelli *et al*., 2006; Harvey‐Samuel *et al*., 2014). This enables SIT‐like applications in further species for which a sterilizing dose of radiation can cause too much collateral damage to somatic cells and/or tissues.

As the phenotypes of these novel constructs are engineered rather than caused randomly by irradiation, molecular biologists can design when and where a lethal gene is expressed. One important outcome is dominant female‐specific lethality, alternatively described as male‐selecting constructs (Heinrich & Scott, 2000; Thomas *et al*., 2000; Fu *et al*., 2007, 2010; Wise de Valdez *et al*., 2011; Ant *et al*., 2012; Labbé *et al*., 2012; Jin *et al*., 2013; Tan *et al*., 2013) (Fig. 1B). Daughters of homozygous transgenic males are not viable, except when reared on diet containing the repressor. Their sons survive, possibly with minor fitness costs. This sex‐specificity can be exploited to enable male‐only release because removing the repressor from the final generation of insects prior to release results in only males surviving, thus achieving sex‐separation by ‘genetic sexing’. Another novel genetic trait, developed in container‐dwelling mosquito species (Phuc *et al*., 2007), has lethality occurring after the immature life stage that is affected by density‐dependent competition mortality and before reaching maturity; adult females are harmful as they bite and transmit disease. Where the juvenile stage of a pest insect causes damage to plants, such a late‐acting trait is less attractive. Variations also include tissue‐specific expression to render female mosquitoes flightless and therefore unable to feed or mate (Fu *et al*., 2010; Wise de Valdez *et al*., 2011; Labbé *et al*., 2012).

An alternative, non‐genetic variant of the SIT, known as the incompatible insect technique (IIT), is being developed in mosquitoes (Bourtzis, 2008). This involves the sustained release of males infected with *Wolbachia*, an intracellular bacterium that interferes with reproduction, rendering matings between released males and wild *Wolbachia*‐free females infertile (Werren *et al*., 2008; Bourtzis *et al*., 2014). Although IIT may be applicable to the integrated management of vectors, as far as we are aware, there are (as yet) no proposed applications of this technology to plant pest insects; one key reason is the need for a highly effective sexing strain or sex‐separation method to enable only males to be released, because any released females could establish the *Wolbachia* infection in the wild population, thereby removing the incompatibility (Bourtzis, 2008).

## Agricultural pest management: mathematical modelling

Starting with the USDA in the 1950s (Knipling, 1955), mathematical modelling has long been used to understand the potential effect of sterile insect methods on an insect population (Alphey & Bonsall, 2014b). Models can address research questions relevant to a particular context, whether the target insect is a plant pest that causes damage when ovipositing, through feeding or by transmitting plant pathogens, or is a vector of human, livestock or wildlife diseases. Those research questions can serve a range of purposes, including helping to understand underlying biological processes, designing appropriate traits, predicting the impact of fitness costs, informing the design and evaluation of experiments, or exploring potential benefits.

A common theme in this work is to combine ecology and genetics. For example, modelling the effects of larval competition and exploring late‐acting lethal phenotypes in mosquitoes predicted that this could be substantially more effective at population control than an early‐acting (e.g. embryonic) lethality or radiation‐induced sterility (Atkinson *et al*., 2007; Phuc *et al*., 2007; Alphey & Bonsall, 2014a). Indeed, if density‐dependent juvenile competition were over‐compensatory, genetic lethality that occurred at an earlier stage, thereby freeing survivors from regulation by intense competition, could push adult insect numbers higher than in the natural uncontrolled population (Yakob *et al*., 2008; Alphey & Bonsall, 2014a). This multidisciplinary approach can be broad; population dynamic models incorporating density‐dependent competition were combined with epidemiological models to investigate the potential effect of releases on a mosquito‐borne disease in a human population (Atkinson *et al*., 2007; Alphey *et al*., 2011a) and linked with bio‐economic and health economic models to estimate the potential cost‐effectiveness of this novel vector control (Alphey *et al*., 2011a). Similar multicomponent modelling approaches could be applied to plant pests, to explore potential for cost‐effective population control to limit crop yield losses.

**Figure 2:**
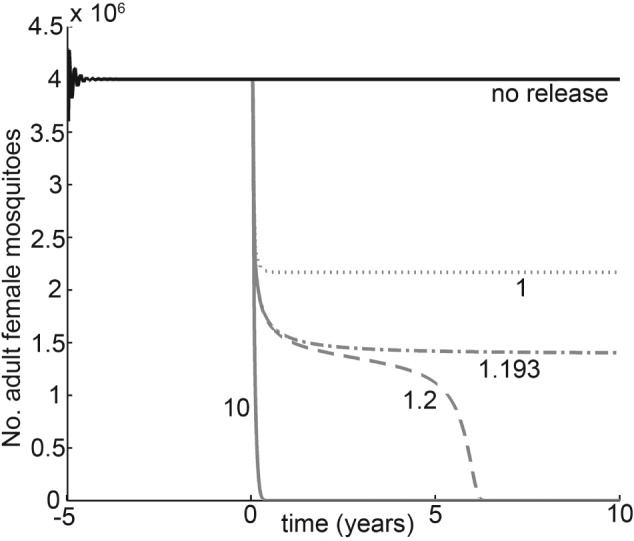
Population dynamics. Insect population over time, with no release (black) or release of ‘sterile’ males homozygous for a bisex‐lethal construct (grey lines) at a release ratio of 1 (dotted), 1.193 (dash‐dot), 1.2 (dashed) or 10 (solid) released males maintained in the environment to the number of wild males at equilibrium. The critical release ratio lies between 1.193 : 1 and 2 : 1. This example is a deterministic, continuous‐time model of a density‐regulated Aedes aegypti mosquito population, illustrating the simulated number of adult females (these are the disease vectors). Model and parameter values as in reported in Alphey et al. (2011a).

In a simple deterministic population dynamic model with density‐dependent regulation (Alphey *et al*., 2011a), pest numbers approach a natural equilibrium, or oscillate around it (Fig. 2). Genetic control using modified males can be incorporated by scaling reproductive growth by the fraction of matings that produce viable progeny (the number of fertile males divided by the total number of males, assuming a well‐mixed, randomly mating population) (Alphey & Bonsall, 2014b). The density dependence term in the formula is adjusted according to whether the genetically‐induced mortality occurs before or after the competition takes effect (Alphey & Bonsall, 2014b). Critical thresholds, or tipping points, are a common feature of models of genetic control (e.g., Atkinson *et al*., 2007; Phuc *et al*., 2007; Alphey *et al*., 2009, 2011b; Alphey & Bonsall, 2014a,b). A ‘release ratio’ or ‘overflooding ratio’ can be defined in various ways, such as the ratio of engineered males to the number of wild males at natural equilibrium to be maintained at constant value through sustained ongoing releases balancing out mortality, or as a fixed proportion of released males to males emerging in the wild. Whatever the precise definition used, there exists a critical ratio. If engineered males are released at a sustained ratio higher than that critical threshold, the population will be eliminated. If the release ratio is below the critical value, the population attains a new, lower, equilibrium density. Such population suppression would be considered a practical success if that new lower equilibrium were below an economic harm threshold for a crop pest. Moreover, this suppression might be of ecological benefit because the species is not totally eliminated from an ecosystem. A population cannot, however, be suppressed arbitrarily close to zero and there is a clear switch from suppression to elimination as the critical release ratio is passed.

The impact of mating competitiveness of released insects can be explored using population models. A key practical result of a mathematical model of genetic control of the mosquito *Aedes aegypti* (L.) (Phuc *et al*., 2007), which transmits viruses including dengue, yellow fever and Zika, suggested that those engineered males would need to achieve 13–57% of matings to achieve sufficient suppression to reduce the disease burden of dengue virus. This model prediction has guided assessment of the performance of the strain in open field trials (Harris *et al*., 2011, 2012). Populations of *Ae. aegypti* have been suppressed successfully in field trials in the Cayman Islands (Harris *et al*., 2012), Panama (Gorman *et al*., 2015) and Brazil (Carvalho *et al*., 2015).

In principle, genetic sterile insect methods could work alone. However, they are more likely to be practical, cost‐effective and sustainable (delaying the evolution of resistance) when used in combination with other approaches.

## Integrating pest management methods

Genetic insect control methods need not be directly aimed at population suppression. The female‐lethal, or male‐selecting, versions could in principle be used to help manage resistance to other control methods. First, consider an example of another plant pest control method using GM technology: insecticidal crops.

Transgenic Bt crops are engineered to express insecticidal toxins derived from *Bacillus thuringiensis*, causing mortality to susceptible insects eating the plant (Tabashnik *et al*., 2013). Effective Bt crops are valuable and there is a strong economic threat from the propensity of insects to evolve resistance. The primary approach used to slow the evolution of resistance is known as the high‐dose/refuge strategy and this is mandatory in some countries. The effectiveness and dominance of resistance to toxins is often dose‐dependent. Commercial crop varieties are designed to express a ‘high dose’ of the relevant Bt protein, so that, if any allele in the population is able to confer resistance, the amount of toxin expressed will be sufficient to kill resistant heterozygotes. If this is achieved, the resistant allele is functionally recessive. Planting high‐dose Bt crops across an entire landscape would likely lead to the rapid spread of resistance because the only individuals that could survive would be homozygous. The ‘refuge’ part of the strategy provides an area of nontransgenic plants (either a conventional variety of the crop or an alternative host plant species) to serve as a safe harbour for susceptible insects. This acts as a source of susceptible alleles and helps to dilute and slow the evolution of resistance by providing susceptible mates for resistant insects so that their progeny are heterozygous and are killed by the toxin (Tabashnik *et al*., 2008, 2013).

In terms of the genetics, consider a resistant allele *r*, which is initially rare. The dominant allele *S* is susceptible to Bt. If the high dose assumption is achieved, only some *rr* individuals can survive a full life cycle on transgenic plants and emerge as adults to mate. In the refuge, most emerging adults will be susceptible *SS*, especially if the *r* allele has fitness costs in the absence of the Bt toxin. If the refuge is located so that the two subpopulations are well‐mixed, most resistant *rr* survivors will mate with susceptible *SS* insects from the refuge. Their resulting *Sr* progeny cannot survive on the Bt crops and so will not pass on the resistant allele to future generations. Unfortunately, a few insect species, such as the economically important pests *Helicoverpa zea* (Boddie) and *Helicoverpa armigera* (Hübner) (both species, confusingly, known as both bollworm and corn earworm), have been identified in the past as ‘moderate dose pests’, where the toxins were unable to kill all heterozygotes (EPA, 1998; Tabashnik *et al*., 2008). Even where its main assumptions appear to hold, the high‐dose/refuge strategy is predicted only to delay resistance and, after two decades of commercially grown Bt cotton and Bt corn (maize), some field‐evolved resistance has now been observed and reported in a variety of insect species. In some cases, this has already led to reduced efficacy of crops, or even crop failures. A comprehensive review is provided by Tabashnik *et al*. (2013).

In economic terms, the high‐dose/refuge strategy is an inter‐temporal trade‐off, sacrificing current value to retain value generated in future (Frisvold & Reeves, 2008). Refuge plants will be damaged, which reduces their yield if they are crops, or reduces the area allotted to crop production if alternative host plants are used. This damage or lost production is tolerated, in return for prolonging the efficacy of the protection afforded by the Bt crops. In principle, a conventional crop refuge could be sprayed with another pesticide (one with no cross‐resistance between its active ingredient and Bt), although doing so reduces its effectiveness as a refuge.

Next, consider combining Bt plants with a female‐lethal genetic insect control programme. The males to be released should carry two copies of the lethal construct and should also be homozygous susceptible to Bt, *SS*. This results in introgression of genes through the male line, with male progeny of released insects inheriting an *S* allele and therefore passing it on to their offspring, at least in the refuge. Viewing this as an alternative source of susceptible alleles, we investigated whether mass‐release of these toxin‐susceptible insects could substantially delay or reverse the spread of resistance to Bt and reduce the need for a refuge (Alphey *et al*., 2007).

**Figure 3:**
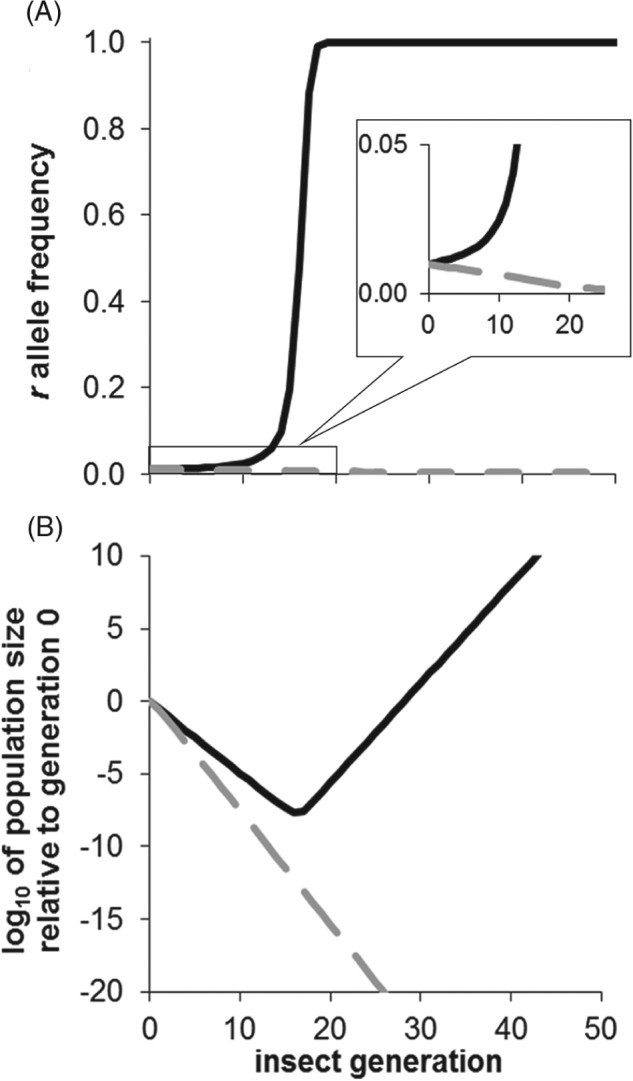
Resistance management. (A) Frequency of the resistant r allele in emerging adults and (B) population size relative to initial size, over time (insect generations) with no insect releases (black lines) and with release of toxin‐susceptible modified males carrying a female‐lethal genetic construct at fixed ratio of 1 : 2 to males emerging in the wild (grey lines). The released insects act in synergy with the Bt crops. This represents a generic pest for which resistance to Bt plants is recessive with partially‐dominant fitness costs, where refuge is 4% of the habitat. Based on a deterministic, discrete‐generation model reported in Alphey (2009).

Simulation models were used by the U.S. Environmental Protection Agency when developing the regulations for Bt crops, and specifying resistance management requirements including minimum refuge sizes and spatial restrictions (Matten *et al*., 2012). We used a population genetic model to investigate the effect of releases of susceptible female‐lethal engineered males on the evolution of Bt resistance over time (Alphey *et al*., 2007). With plausible parameter values, the *r* allele can spread (Fig. 3A) and spreads faster with a smaller refuge. Generally, a frequency of 0.5 is an indicator of a serious problem because resistance frequency increases rapidly once *r* becomes the more common allele (Carrière & Tabashnik, 2001). With a very modest ratio of released males to males emerging in the wild (sustained over time), our models predict that releases can slow or reverse the spread of resistance to Bt crops. Although a typical suppression programme might aim for a ratio of 10 : 1 or more (Dyck *et al*., 2005), resistance management programmes could see an effect with ratios as small as 1 : 5 (i.e. where one‐sixth of matings are with a modified male). Engineered insect releases allow an equivalent level of resistance management with a smaller refuge. If the initial *r* allele frequency is higher, more released males and/or a larger refuge are required to achieve a given effect. The release numbers and refuge size can be traded off, which would be at least in part an economic decision (Alphey *et al*., 2007).

The objective is to protect crops from damage. The resistant allele frequency is not important in itself. What really matters is whether the pest population is too large. Linking the population genetics model with a simple population dynamic model of a pest exhibiting exponential growth (if uncontrolled), we predicted that insect releases always improve population control (compared with Bt crops alone, with the same refuge size) because of the combination of resistance dilution and suppression (Fig. 3B). This difference may not be easily detectable in the early generations, but the outcomes diverge significantly when resistance spreads beyond 0.5 allele frequency without releases (Alphey *et al*., 2009).

Sterile insect methods can be used remedially where resistance has become widespread. A refuge cannot exceed 100% (i.e. plant no Bt crops at all), and by itself would rely on fitness costs to cause the *r* allele to decline. Higher release ratios can rapidly reduce the *r* allele frequency at the same time as directly reducing the population size. Releases over Bt crops can be used to reverse the spread of resistance, although the reduction of resistance to acceptably low levels needs an appropriate combination of release ratio and refuge size (Alphey *et al*., 2009).

Optimal control theory has been applied to a generic high‐dose refuge scenario, exploring resistance management decisions for planting of Bt crops and refuges each season. A model using dynamic programming methods investigated the potential for fitness costs of resistance to enhance the delaying effects of the refuge, where the biological nature of those costs was a reduction either in fecundity or in relative competitive ability. Where the fraction of the landscape allocated to Bt crops is decided optimally, a resistance‐related fecundity penalty allows for the planting of larger areas of Bt crops than equivalent costs that reduce the ability of resistant insects in density‐dependent competition (Hackett & Bonsall, 2016). Work is ongoing to extend this approach to incorporate the genetic control of plant pests along with insecticidal crops or sprays.

Female‐lethal genetic control releases are potentially a useful addition to the resistance management toolkit. Theory predicts this combined approach could be effective at very low release ratios, much lower than would be needed for suppression or eradication by SIT, and permit much smaller refuges than would otherwise be needed and are typically mandated (Alphey *et al*., 2007). Males engineered with female‐lethality acting in synergy with Bt crops could be effective in a wide range of ecological and genetic scenarios (Alphey *et al*., 2009). Building on this theoretical work, genetic strains of diamondback moth with the female‐lethal trait have been developed (Jin *et al*., 2013) and tested for fitness costs with population‐level effects (Harvey‐Samuel *et al*., 2014). Proof of principle of population suppression combined with resistance dilution has now been demonstrated in field‐cage experiments with experimental Bt broccoli (Harvey‐Samuel *et al*., 2015) and work is progressing towards open field trials to evaluate performance in an agricultural habitat (Cornell University, 2016; Oxitec Ltd, 2016).

This example of single‐toxin Bt crops is one illustration of using engineered insect releases to help manage resistance to another control method. The idea is also applicable in principle to other approaches, such as Bt biopesticide sprays or synthetic chemical insecticides.

Successful sterile insect release programmes have been implemented as area‐wide operations, usually with involvement and investment from local or national government and/or international government and agencies (Dyck *et al*., 2005). Growers' associations and similar bodies may also be involved including, for example, by contributing to programme funding through levies. The release of GM insects, as described above, for concurrent suppression of insect populations and dilution of resistance to another control method (such as Bt crops), could follow this precedent. The economics of particular pest and crop species would have to be considered in the context of any current regulation (e.g. mandatory refuge sizes for Bt crops) and environmental harm from current practices (e.g. insecticidal sprays), to assess whether such insect releases are likely to be of benefit to the various participating stakeholders.

## Intrinsic resistance dilution

We can take this concept of using transgenic insects to manage resistance a step further. What if heritable resistance were to arise to the lethality of the genetic construct itself? Such resistance has not been reported in any species of engineered insects, whether in trials or at an earlier stage of technology development, although a hypothetical resistant gene can be described and modelled (Alphey *et al*., 2011b). Key features of a putative resistance allele (*R*) are: the effectiveness of the resistance (what fraction of *RR* individuals bearing a lethal allele can survive to maturity?); the dominance of resistance (the lethal‐surviving proportion of *SR* genotypes, relative to *RR* homozygotes); and the magnitude and dominance of any fitness costs of the *R* allele in individuals that do not carry the lethal construct. With good quality control, released males can be assumed homozygous susceptible to the lethality of the genetic construct (*S* alleles) and do not carry any mutation or variant conferring resistance (*R*) that might be present in the wild population. In a similar vein to the Bt resistance management described above (Alphey *et al*., 2007, 2009), any progeny of released males will inherit one copy of the susceptible *S* allele, as well as one copy of the lethal genetic construct, which provides resistance dilution. The *S* alleles of released males are inherited through their male progeny where the construct is female‐specific, and are also passed on via any progeny that survive the lethal effect (i.e. resistant phenotype). So, if resistance is not recessive (some *SR* individuals can survive), a bisex‐lethal mechanism will also have this inherent resistance dilution potential (Alphey *et al*., 2011b).

There are two opposing causes of selection pressure. Progeny of released engineered males inherit: (i) the lethal allele, which favours resistance and (ii) a susceptible *S* allele, which dilutes resistance.

The fitness advantages and disadvantages of resistant *R* alleles are negatively frequency dependent. Resistance is only beneficial in insects bearing the lethal construct, and the costs of resistance are mainly manifested in individuals that do not carry the lethal allele. The overall selection pressure is also dependent on the number of transgenic insects in the population and therefore on the release ratio deployed. We created a frequency‐dependent population genetic model, with the number of released males kept in fixed proportion to the number of males in the current generation of the target population, aiming to explore how these competing forces play out (Alphey *et al*., 2011b). The model displays a complex array of possible outcomes. In summary, for some putative resistant alleles, the built‐in dilution can be sufficient to drive any resistant allele extinct before it reaches observable frequency. More challenging resistant alleles, those with greater effectiveness against the lethal and lower fitness costs, can potentially spread through the population to an equilibrium level, although this will not necessarily have a major impact on the effectiveness of controlling the pest population (which depends on the reproductive rate of the species).

Further work has extended this research idea to explore the effects of spatial structure using a two‐deme metapopulation (Watkinson‐Powell & Alphey, 2017). A nontarget population, linked by dispersal with the target population into which modified insects are released, can act either as a source of susceptible alleles (acting as a kind of refuge) or as a source of resistant alleles depending on the fitness properties of the resistant allele. The rate of dispersal also influences the outcomes. As a result, the presence of a nearby nontarget population could have a range of impacts from significantly hindering the control programme to significantly enhancing it. Further work has also demonstrated qualitatively similar findings to the original proportional‐release model under the alternative, and arguably more practical assumption, that a fixed number of modified males is released into each pest generation (Thompson, 2015).

## Gene editing

Heritable genetic ‘sterility’ is not the only genetics‐based method being developed to control insect populations (Alphey, 2014; Burt, 2014). Recent advances in genetic modification have focussed on techniques of gene and genome editing. Molecular methods, including CRISPR (‘clustered regularly interspaced short palindromic repeats’) approaches, have been developed with the aim of precisely modifying genes (Esvelt *et al*., 2014; Kim & Kim, 2014). These techniques have the potential to drive genetic constructs through a population, incorporating ‘gene drive’ mechanisms that confer greater‐than‐Mendelian inheritance even if the construct has fitness costs.

These gene‐editing approaches have been developed in mosquitoes either to suppress vector populations, by affecting female fertility (Burt, 2003; Deredec *et al*., 2008; Hammond *et al*., 2016), or to modify a population, by spreading a trait that affects the ability to harbour pathogens (Gantz *et al*., 2015). Gene‐editing approaches could also be used to suppress agricultural pests and/or manage resistance; for example, CRISPR gene editing has been used in a functional study to identify suitable gene targets in diamondback moth (Huang *et al*., 2016). However, considerable technical, ecological, regulatory and social engagement work remains to be carried out as these approaches move towards scalable field implementation.

## Interdisciplinary research: theoretical, laboratory and field

Developing genetic approaches to insect control through to field applications is an interdisciplinary endeavour. Theoretical analyses such as those described in the present review are part of a much bigger picture, a composite of varied elements that must work together to achieve real change. Laboratory science is crucial for the creation of appropriate strains, particularly molecular biology and insect genetics. Applying this technique successfully to populations in nature is largely an exercise in applied ecology. For example, how many insects are in the target population? This is hard to measure, although it is a key element of the effective release ratios that will be achieved, and so influences the impact, duration and cost of a control programme. How might the effects of identified fitness costs scale up to population level? Insect behaviour is important; where do they mate and lay their eggs, and how far can they disperse? Released engineered males must be able to reach a significant proportion of females in the target population and be reasonably competitive for mates when they find them. Evolutionary biology and behavioural ecology must be understood, for example, to ensure that mass‐reared insects retain appropriate mating behaviours, and to inform future resistance management strategies for self‐sustaining genetic traits that will be designed to persist in the environment.

A variety of performance measures are critical to success, including a lethal phenotype's conditionality (do transgenic insects survive on the antidote‐containing diet?), lethality (do close to 100% of target insects die in absence of the repressor?) and sex‐, tissue‐ or stage‐specificity (does a female‐lethal construct produce any detrimental fitness effects in males?), in addition to the longevity, flight ability, dispersal, mating behaviour and competitiveness of the released males.

Candidate lines are selected and tested, assessing all these crucial performance measures in a stepwise series of experiments and trials progressing from test tube, through small cages, then large cages (semi‐field conditions), to open release. Technology development, pilot studies and control programmes involve other disciplines beyond science; there are also regulatory, social and ethical dimensions with respect to implementing this approach (Lavery *et al*., 2008; Esvelt *et al*., 2014).

## Policy and regulation of genetic insect control

Policy and regulations surrounding genetic insect control have developed and expanded in the last few years and continue to receive attention (e.g. House of Lords Science and Technology Committee, 2015). Based on existing environmental risk legislation, in most jurisdictions that have regulatory frameworks for these, the deliberate release of genetically modified insects requires proportionate assessment to ensure that wider biodiversity and/or human health is not adversely affected. Simultaneously, the benefits of suppressing agricultural pests, reducing harm and improving plant yields impinge on cost–benefit analysis in using particular control technologies.

Across the European Union, Directive 2001/18 requires Member States to evaluate risks of releasing GM organisms (GMOs, whether plants, vaccines or animals). This is a risk (cost) based approach to the deliberate release of GMOs, which is based on the use of recombinant DNA technology (i.e. genetic modification) as the trigger for regulation. Contrasting regulatory processes exist. In Canada, for example, the legislation for ‘plants with novel traits’ outlines that the phenotypic effects (novel traits) of the plant are the basis for regulation, which is a more ‘product‐based’ approach to triggering an environmental risk assessment (Canadian Food Inspection Agency, 2016).

For GM insects, several guidance frameworks have been produced in recent years. The European Food Safety Authority (2012) published a regulatory framework for GM animals and the World Health Organization (WHO/TDR and FNIH, 2014) issued guidance for the testing and regulation of GM mosquitoes. Both of these asserted that a tiered approach from laboratory studies (focussed on the molecular biology and simple ecological processes) through to contained or confined field trials to commercial implementation should underpin environmental risk assessment in support of the development of GM insect technologies. At each point, risk assessment, risk management and risk communication ensures the validity of the emerging technology.

Unlike plants, where genetic modifications are compared with an unmodified (conventional) plant, defining harm and identifying appropriate comparators for genetically modified insect pests requires more nuanced approaches. Proportionate to the technology and logically consistent with other pest intervention methods, appropriate ways of assessing the environmental risk might focus on changes in crop yields or other indirect measures of assessment (e.g. scale of insect damage). Developing regulations that consider the risks in the context of the potential benefits, and not as an isolated risk (or worse a conflated hazard), requires further consideration about the implementation of all types of area‐wide control such as who benefits and who pays for these public goods.

Public acceptance of GM technologies has varied across products (crops, insects, vaccines and insulin), as well as between countries and communities, and public engagement concerning GM insects will continue to be an important issue for developers and regulators (House of Lords Science and Technology Committee, 2015).

## Concluding remarks

The need for new innovations to deal with emerging agricultural pests and diseases has never been greater, and this is an interesting time for the science and research of genetic control of insects. Some of the GM technologies described in the present review are already being proven in the field. The next wave of molecular methods is being applied to disease‐transmitting mosquitoes and this is beginning to reach over to agriculturally important species. Attention is being given to regulatory aspects to enable the safe and appropriate implementation of these biological, genetics‐based strategies. Collectively, these developments advance the prospects for realizing tremendous agricultural and socio‐economic benefits.

## Further information

This review is part of a larger project. Further information on our research and science policy work on genetic insect control can be found on the Mathematical Ecology Research Group website: https://merg.zoo.ox.ac.uk/projects/genetic-insect-control. Our outreach activities, including a mosquito control computer game are available at: https://merg.zoo.ox.ac.uk/outreach. Our science policy work can be seen at: https://merg.zoo.ox.ac.uk/science-policy.
